# Becoming biomedical laboratory scientists: exploring students’ professional development through communities of practice

**DOI:** 10.1186/s12909-026-10801-x

**Published:** 2026-11-11

**Authors:** Amrah Heikkinen, Jenny Flygare, Max Scheja, Klara Bolander Laksov

**Affiliations:** 1 Department of Education, Stockholm University, Stockholm, Sweden; 2 Department of Teaching and Learning, Karolinska Institute, Stockholm, Sweden

**Keywords:** Biomedical laboratory scientist, Professional development, Belonging, Communities of practices

## Abstract

**Background:**

Professional development is a central component of healthcare education, yet little is known about how Biomedical Laboratory Scientist (BLS) students develop their understanding of the profession throughout their education. This study explored Biomedical Laboratory Scientist students’ professional development from a communities of practice perspective (CoP), examining how knowledge, practices, values, and professional understanding were described across three years of undergraduate education.

**Methods:**

A qualitative, cross-sectional study was conducted with 24 BLS students from the first, second, and third years of a Swedish university programme. Students participated in semi-structured peer interviews using a shared interview guide and recorded their discussions. Data was analyzed using qualitative content analysis, informed by Wenger's Communities of Practice framework, with imagination, engagement, and alignment serving as analytical lenses.

**Results:**

Professional development can be understood as an evolving process shaped by educational experiences, clinical placements, peer dialogue, and personal reflection. Early in the programme, the profession was primarily imagined through prior expectations and limited experience. As education progressed, engagement in laboratory practice and clinical learning broadened the understanding of professional responsibilities. Towards the end of the programme, professional values became increasingly aligned, while future roles within the BLS community were being negotiated and explored.

**Conclusions:**

This study explored BLS students’ professional development across three years using Wenger’s Communities of Practice perspective. Findings indicate that professional development was an evolving process involving imagination, engagement, and alignment, rather than a linear progression towards professional belonging. Educational and clinical experiences supported developing professional knowledge, practices, and understanding, while also prompting critical reflection and, for some students, professional distancing. These processes support competence, development and application in practice, contributing to quality and patient safety.

## Introduction

Professional development is an important component of healthcare education because students progressively develop the knowledge, skills, values, judgement, and practices required for professional practice. Although professional development continues throughout working life, its foundations are established during professional education, when students move from predominantly academic learning towards increasingly complex participation in healthcare practice [11, 29, 30]. Research across healthcare professions, including nursing, medicine, and physiotherapy, suggests that this development is shaped not only by formal curriculum and competence assessment but also by clinical learning, supervision, feedback, peer interaction, reflection, and participation in authentic professional practice [13–15]. Professional development during education can therefore be understood as an evolving process through which students integrate theoretical knowledge with practical skills, professional values, judgement, and responsibilities within healthcare contexts. This process overlaps with professional identity formation (PIF), which concerns how students come to understand themselves as members of a profession. The emphasis of this paper, however, is on social and collective learning within the community of BLS professionals who contribute to healthcare through laboratory investigations that inform diagnosis, treatment monitoring, disease management, and patient safety. Despite this important role, our literature search identified limited research specifically examining professional development during BLS education.

### Literature review

Given the limited research identified on professional development in BLS education, it is necessary to draw on research from other health professions. Research in nursing and BLS demonstrates that professional competence extends beyond technical performance to include communication, ethical practice, interpersonal relationships, clinical reasoning, reflection, and professional behaviors [13, 14, 19]. Similarly, physiotherapy research highlights clinical education, supervision, interaction with practitioners, feedback, and participation in practice as important contexts for developing professional capabilities [15]. According to the research, the educational value of practice-based learning appears to depend on the opportunities students have to participate, interact, receive feedback, and reflect. These opportunities can support professional development by helping students connect theoretical knowledge with practical decisions, examine their own performance, and develop an understanding of professional values and responsibilities (ibid.). Likewise, medical education research indicates that clinical and educational experiences contribute to students’ development of professional knowledge, behaviors, and responsibilities [23]. Together, this literature suggests that professional development is not simply a linear accumulation of predefined competencies but involves progressively integrating and applying knowledge and practices through educational and professional experiences.

This perspective is also relevant to BLS education. BLSs contributes to healthcare through laboratory investigations that inform diagnosis, treatment monitoring, disease management, and patient safety. Professional practice in BLS integrates scientific and technical expertise with quality, safety, communication, and professional responsibility. A Delphi study further highlighted trustworthiness, self-awareness, and professionality as key competencies, underscoring that BLS competence extends beyond technical laboratory procedures [27]. Recent work on BLS curricula similarly emphasizes broad professional competencies alongside technical knowledge and skills in response to changing healthcare and laboratory practice [13, 17, 18].

Despite this broader understanding of competence, the BLS education literature identified in our research overview addressed competency requirements, curriculum content, clinical practicum, assessment, and students’ readiness for professional practice [1, 7, 27]. Such research is essential for ensuring educational quality and patient safety. However, competency-oriented approaches primarily address what students should know and be able to do, while providing less insight into how students progressively develop, integrate, and understand these capabilities during their education. Examining professional development may therefore provide a complementary perspective on BLS education.

Clinical placements and other practice-based experiences may be particularly important in this process. Through participation in laboratory practice, students encounter professional routines, quality and safety requirements, collaboration with colleagues, and the consequences of laboratory work for patient care. Such experiences can provide opportunities to contextualize theoretical knowledge and develop practical understanding. Evidence from nursing, physiotherapy, and medicine indicates, however, that the value of practice-based learning depends on the quality of supervision, feedback, interaction, participation, and reflection [13–15, 23]. Professional development should therefore not be viewed as an automatic or uniform consequence of progressing through an educational programme or completing clinical placements. Students may enter education with different expectations, encounter different forms of professional practice, and progressively reinterpret their understanding of professional knowledge, practices, values, and responsibilities. To better understand professional development during BLS education, it is thus of interest to explore students’ experiences at different stages of their education.

Professional learning is also inherently social [29] (BLS practice is embedded within laboratory teams and the wider healthcare system, making collaboration and understanding of professional roles relevant dimensions of development. Professional development therefore involves learning not only how to perform laboratory procedures but also of how laboratory knowledge and practices contribute to broader healthcare processes.

Despite the importance of professional development in preparing BLS for professional practice, limited research has examined how this development unfolds across undergraduate BLS education. In particular, little is known about how the profession at different stages of education describes the developing of professional knowledge, practices, values, and understanding. Therefore, this study explored BLS professional development across three years of undergraduate education from a CoP perspective, examining how knowledge, practices, values, and professional understanding were described at different stages of their education.

### Context of biomedical laboratory scientist education in Sweden

In Sweden, becoming a BLS requires completion of a three-year university programme leading to licensed professional qualification and a Bachelor of Medical Science degree with major in Biomedical Laboratory Science. The profession includes two main areas of specialization: laboratory medicine, focusing on analysis of biological specimens, and clinical physiology, focusing on assessment of physiological functions. Despite differences in practice, both areas contribute essential information for diagnosis, treatment decisions, and monitoring of disease progression. The BLS education examined in this study provides an important context for exploring professional development because students’ progress through different stages. During the first year, students study together regardless of specialization, developing foundational knowledge and an initial understanding of the profession. During the second and third years, students enter their chosen specialization and participate in increasingly extensive clinical placements. This transition from university-based learning to workplace participation provides opportunities for students to negotiate professional development competence, and experience membership within professional communities [25].

### Communities of practice as a theoretical framework

To examine how BLS students develop professionally, this study draws on Wenger’s [29, 30] theory of CoP, which conceptualizes learning as a social process in which individuals develop knowledge and development through participation in shared practices. From this perspective, becoming a professional is not simply a process of acquiring skills, it is a process of becoming a member of a professional community through interaction, participation, and negotiation of meaning. Wenger [29, 30] proposes three modes of identification that describe how the profession locates itself within a CoP: imagination, engagement, and alignment.

Imagination refers to how professions construct possible professional development by drawing on experiences, role models, and representations of the profession. For the BLS profession, this may involve developing an initial understanding of what it means to become a laboratory professional. Engagement represents active participation in professional practices where professionals interact with peers, educators, and clinical professionals while developing practical competence and negotiating professional meanings. Alignment involves connecting professional actions and values with broader professional expectations, allowing the learners to position themselves as legitimate members of the profession.

These modes provide an analytical framework for examining professional development as a trajectory rather than a fixed achievement. Individuals may move between imagining the profession, participating in professional practices, and aligning themselves with professional values throughout their education. A further important concept within CoP is the relationship between participation and reification. Participation refers to involvement in meaningful professional activities, whereas reification describes how abstract concepts become understood through concrete experiences [29, 30]. For example, students may initially encounter quality assurance as a theoretical concept but gradually understand it as a professional responsibility through clinical practice. These processes are interconnected: participation gives meaning to professional concepts, while reified knowledge- such as, creation of artefacts, concepts, or representations that make shared meaning visible and durable within a CoP- shapes future participation.

Although CoP provides a useful framework for understanding professional development as socially situated learning, Wenger’s ideas have also been subject to critique. Critics have argued that CoP may underemphasize professional agency and the influence of power and structural conditions on participation [6, 26]. Communities may also involve unequal access, exclusion, and competing interests rather than being cohesive or supportive spaces for learning [10, 26]. These limitations are relevant to students whose opportunities for participation are shaped by their educational position and clinical contexts. Nevertheless, we chose to use CoP in this study for its emphasis on participation, meaning-making, and professional communities which provides a framework for examining professional development as an evolving and socially situated process.

By applying CoP, this study moves beyond describing whether professional development occurs and instead examines how BLS students construct their professional development over time. Specifically, our study objective is to explore how students understand their profession at the beginning, midpoint, and conclusion of their education. Through examining this developmental trajectory, the study addresses an important gap in healthcare education research by expanding understanding of learning trajectories within an underexplored healthcare profession.

## Method

### Study design

This study employed an empirical, cross-sectional qualitative design to explore how BLS students understand their profession and its professional development at different stages of their education. This implies a focus on following the group at an aggregate level and examining differences throughout the progression of the programme, rather than following individual trajectories. A social constructivist perspective informed the study, as knowledge and professional development are viewed as socially constructed through dialogue and shared experiences [3]. This perspective was considered appropriate because the study sought to understand how students construct meaning around becoming BLS professionals rather than measure predefined outcomes.

### Setting and participants

The study was conducted within the three-year BLS programme at a large Swedish university. To capture students' understandings across the educational trajectory, participants were recruited from the first, second, and third years of the programme. Following approval from programme leadership, the first author visited scheduled teaching sessions to introduce the study. Students received written and verbal information about the study, including its purpose, procedures, voluntary nature, and ethical considerations. Information sheets and informed consent forms were distributed during these sessions, allowing students time to consider participation before providing written consent. Twenty-four students volunteered to participate from the 115 students attending the information sessions. The sample comprised eight students from each year of the programme. Within each cohort, six students were enrolled in the laboratory medicine specialization and two in clinical physiology. This is representative of the larger student population on the programme. The Year 1 cohort included four women and four men, whereas the Year 2 and Year 3 cohorts each included six women and two men. As participation was voluntary, the sample may reflect self-selection, with students who were more engaged or interested in reflecting on their educational experiences being more likely to participate. Nevertheless, including students from all three years enabled comparisons across different stages of professional development.

### Data collection

Based on the study objective and theoretical perspective with CoP theory five open-ended questions were created addressing students’ motivations for entering the BLS profession, perceptions of themselves as future BLS professionals, professional development during their education, perceived challenges, and the role of accuracy in their future profession. Previous research has indicated that power imbalance between researcher and interviewee may be solved through a peer-interview format [8, 16, 21]. We therefore decided to approach data collection through peer-led semi-structured interviews, in which students interviewed one another rather than being interviewed by a researcher to enable a participatory approach in line with our theoretical framework.

Student-led peer interviews were conducted separately within each study year, with eight students per year organized into four interview groups. Participants received oral and written guidelines outlining the interview purpose and suggested progression in Swedish (see Appendix 1.) No formal interviewer training was provided beyond these guidelines, and no researcher was present at the interviews. The formation aimed to support reciprocal dialogue and open reflection on personal experiences and professional aspirations. The semi-structured format ensured consistency across interviews while allowing participants to elaborate on issues they considered important.

All participants received information about the purpose of the study, the right to withdraw and sign informed consent forms. All data were anonymized, and participants were assigned numerical identifiers corresponding to the groups in which they participated. Most interviews were conducted in pairs, although one Year 1 interview included four participants. Interview duration increased across the programme, averaging approximately 16 min in Year 1 and 38 min in Years 2 and 3, indicating greater opportunities for reflection in later stages of education. Interviews were audio-recorded with participants’ consent and transcribed verbatim. Audio files, transcripts, and consent forms were securely stored in accordance with institutional data protection procedures. The complete interview guide has been translated into English and provided in Appendix 1.

### Data analysis

The analysis followed an iterative process using deductive qualitative content analysis [12]. Before transcription, the recordings were listened to repeatedly to facilitate familiarity with participants' language, interactions, and conversational context [5]. All authors listened to the interviews, and AH transcribed them. Because the interviews were conducted between peers, transcripts preserved the conversational flow rather than separating responses into individual statements. The material was analyzed deductively using Wenger's CoP framework [9, 29, 30]. The analysis focused on the three modes of identification (imagination, engagement, and alignment) to explore how students developed and negotiated professional development throughout their trajectories of becoming, belonging, and developing as BLS. Imagination concerned students’ constructions of future professional roles, workplaces, and pathways; engagement referred to participation in practices and relationships across educational and workplace settings; and alignment captured the extent to which students coordinated their actions, priorities, and perspectives with the expectations, practices, norms and goals of relevant communities. These dimensions were treated as distinct but interrelated aspects of professional development. Alignment was distinguished from professional belonging, which concerns identification with or perceived membership in a professional group. The framework enabled an interpretive analysis of how students negotiated professional development across different stages of their education while remaining grounded in the empirical data. An illustrative coding matrix is provided in Table 1. During the analysis process, data that did not help in understanding our research questions was excluded.

**Table 1:** Illustrative coding matrix. An illustrative coding matrix is provided in Supplementary Table 2 to demonstrate the analytical progression from participants’ interview accounts to meaning units, condensed meaning units, descriptive codes, and interpretation through Wenger’s three CoP modes. The examples illustrate how imagination, engagement, and alignment were applied as deductive analytical categories while remaining grounded in participants’ accounts of professional development

Interview excerpt	Meaning unit	Condensing meaning unit	Descriptive code	CoP analytical category
“The BLS is quite good at lab work, and this is important if someone wants to do research.” – Year 1, Group 1	The participant associates BLS work with laboratory practice and research	BLS is initially understood through laboratory work and research	Understanding the profession through laboratory practice	Imagination
“It was the closest you could get to becoming a doctor. I wanted to become a doctor, but I didn’t get into the medical programme, and BLS was the closest option.” – Year 1, Group 1	The participant understands BLS in relation to an aspiration to become a doctor	BLS is initially imagined in relation to another healthcare profession	Imagining BLS through comparison with another healthcare profession	Imagination
“I want to become a biomedical laboratory scientist because I love working in the lab.” – Year 2, Group 3	Interest in laboratory work influences the participant’s understanding of becoming a BLS	Laboratory work is viewed as central to the future profession	Imagining the future profession through laboratory work	Imagination
“I'm such a visual person, and I visualize a lot of things. I can see myself in a lab environment. I picture myself in a lab wearing a white coat, with my glasses on, surrounded by a lot of machines, doing a lot of stuff.” – Year 2, Group 3	The participant visualizes the future professional environment and role	The future profession is imagined through the laboratory environment and professional practices	Visualizing future professional practice	Imagination
“I have always thought that I want to work in healthcare. I want to help people and contribute to society, I think.” – Year 2, Group 4	The participant connects future professional work with helping people and contributing to society	The future profession is imagined through contributing to healthcare and society	Imagining professional practice as contribution to society	Imagination
“I want to apply to the police and work there.” – Year 2, Group 1	The participant imagines a future career outside BLS and healthcare	Professional aspirations extend beyond the BLS profession	Imagining alternative professional pathways	Imagination
“I've always enjoyed diagnostics and detective work with anatomy and diseases and stuff.” – Year 3, Group 3	The participant connects BLS work with diagnostic investigation	Professional development is imagined through contributing to diagnosis	Imagining diagnostic professional practice	Imagination
“You should be critical to evaluate [laboratory] results in a meaningful way, to be able to perform an analysis according to a template, maybe.” – Year 1, Group 1	The participant recognizes the need to critically evaluate laboratory results	Laboratory practice requires critical evaluation as well as technical procedures	Developing critical analytical practice	Engagement
“I want to become a doctor. I want to get into the medical programme to continue studying, but I find the subject itself interesting. Studying it seems enjoyable, but perhaps not the profession itself to the same extent.” – Year 1, Group 2	The participant engages with the subject matter while expressing less interest in the BLS profession itself	Educational engagement with BLS-related learning is stronger than occupational identification with the profession	Engaging with the subject while questioning occupational identification	Engagement
“At the beginning of the education it’s almost all theory… we found out more now in the second year, and especially we will find out [more] during the work placement.” – Year 2, Group 3	Practical experiences help the participant understand what BLSs do	Clinical experience connects theoretical learning with professional practice	Learning through clinical practice	Engagement
“We have gained better clarity, like what is important as a BLS… quality assurance, accuracy, analytical skill, and so on.” – Year 2, Group 2	The participant develops an understanding of important professional requirements	Professional expectations become clearer through education	Developing professional knowledge and expectations	Engagement
“I think, or rather, something that you may have already encountered a little, but that you will encounter even more later, is that the profession is so unknown. When you talk to people, they ask, ‘What are you going to study?’ I say, ‘I’m going to study biomedical laboratory science,’ and they are like, ‘Excuse me?’ because no one knows what it is.” – Year 2, Group 2	The participant describes the BLS profession as relatively unknown and becoming more familiar through education	Education provides increasing familiarity with a profession that is initially unfamiliar to both participants and others	Developing familiarity with an unfamiliar profession	Engagement
“I've grown a lot… when it comes to responsibility and being analytical all the time. Not just swallowing things, but being critical and considering… And then, I'm starting to become stress-resistant.” – Year 3, Group 3	The participant describes developing responsibility, critical thinking, and coping with professional demands	Educational experiences contribute to broader professional capabilities	Developing professional capabilities	Engagement
“Nothing is out of the question as a BLS. You can continue to study something else if you want… it’s kind of part of a pathway, and not the end goal, as a BLS.” – Year 1, Group 2	The participant views BLS education as connected to broader professional possibilities	BLS is understood as part of a broader professional pathway	Connecting BLS development with future possibilities	Alignment
“I hope that I will be a good BLS and suited to the profession. I think it’s something that comes with time, and now that I have more clinical training, I’m learning increasingly and it’s becoming more natural.” – Year 2, Group 4	The participant connects increasing clinical experience with becoming more suited to professional practice	Professional development and belonging develop through increasing experience	Developing alignment through professional experience	Alignment
“It feels like there is a risk that there aren’t that many career opportunities. You become a BLS and get stuck, and then you might remain a BLS for 30 years. I’m just like, okay.” – Year 2, Group 2	The participant questions whether BLS provides sufficient opportunities for career development and movement	Perceived limitations in career opportunities create uncertainty about long-term professional alignment	Questioning career possibilities within the profession	Alignment
“Or that we are replaceable, so to speak. But I don’t really think we are. It needs to be made clearer that we actually have expertise that other healthcare professions do not have.” – Year 2, Group 2	The participant reflects on the distinct expertise and value of the BLS profession in relation to other healthcare professions	Professional alignment involves constructing and recognizing the distinctive contribution of BLS expertise	Negotiating professional distinctiveness and value	Alignment
“I feel that this is probably my biggest concern, really—that our profession will become so automated that it will become boring because you do so little yourself… But there are probably areas where this won’t happen. And then there is always the research side, where there are endless possibilities.” – Year 2, Group 3	The participant evaluates whether future automation is compatible with valued aspects of BLS work while identifying research as an alternative professional possibility	Perceived changes in professional practice influence how the participant evaluates future possibilities within BLS	Negotiating alignment with future professional change	Alignment
“In the beginning, you hardly knew what a BLS did other than analyzing samples. Now you know all… the different clinical laboratories that exist, and which one is more to your taste than another.” – Year 3, Group 1	Increased exposure broadens understanding of BLS roles and possible areas of practice	Greater professional knowledge enables consideration of different professional pathways	Connecting professional knowledge with future practice options	Alignment
“I don’t actually see myself as a BLS in the future. I thought we would have more autonomy in the workplace, that we would be able to do more, perhaps have more involvement in reporting results, and have more than just one method that you basically perform, or two that you alternate between. I thought we would be able to do everything, which in theory we should be able to, but in practice you end up performing one or two methods, and that didn’t really appeal to me.” – Year 3, Group 3	The participant compares expectations of professional autonomy and variety with experienced workplace practice	A discrepancy between imagined and experienced practice leads to reduced identification with the BLS profession	Reassessing professional alignment through workplace experience	Alignment
“With this programme, it gives you such a solid foundation for so many different things—it opens so many doors… I really enjoy working with that level of precision, where you become an expert in a specific area.” – Year 2, Group 3	The programme is viewed as providing a foundation for different professional possibilities and specialization	Education connects professional learning with future specialization and development	Aligning education with professional possibilities	Alignment

### Trustworthiness

To enhance credibility, the analytical process involved continuous discussions within the research team throughout coding, categorization, and interpretation. The team included both BLS researchers (JF and AH) and researchers from other disciplinary backgrounds (MS and KBL), allowing interpretations to be challenged from both insider and outsider perspectives. This interdisciplinary dialogue strengthened reflexivity and reduced the risk of profession-specific assumptions influencing the findings.

## Results

This section presents findings related to the three modes of identification within CoP: imagination, engagement, and alignment. The findings are illustrated through selected quotations from peer interviews, presented chronologically across Years 1–3 and across both BLS specializations. The three CoP concepts represent an analytical interpretation of participants’ experiences rather than a sequential developmental pathway shared by all students. The three modes of identification identified in participants’ accounts are summarized in Table 2

**Table 2:** A possible representation of BLS students’ professional development emerging from participants’ accounts. This table presents an interpretive framework developed from the data. It represents one possible pathway of professional development suggested by participants’ experiences and should not be understood as a fixed or universal progression. The three stages illustrate patterns identified within this sample through the CoP identification modes: imagination, engagement, and alignment. Importantly, participants’ accounts also demonstrated variation and movement within and across these modes. Increased knowledge and engagement did not necessarily result in stronger identification with BLS; for some participants, greater exposure led to questioning, renegotiating, or distancing themselves

Stag	CoP identification mode	Emerging professional development
Year 1-Beginning awarness	Imagination of being a BLS	Participants’ initial perceptions of the profession were often associated with natural science, laboratory environments, and practical laboratory skills. The profession was primarily imagined through technical work and participation in laboratory activities. For some participants, however, BLS was initially imagined in relation to other professions or as one possible step within a broader career pathway rather than as a clearly defined professional destination
Year 1-Beginning awarness	Imagination of being a BLS	Participants began developing foundational professional knowledge. Early engagement involved learning theoretical concepts and recognising the importance of scientific reasoning, critical evaluation, and interpretation of laboratory results. At this stage, participants could value the educational content while expressing stronger aspirations towards other professions, indicating that interest in the subject did not necessarily correspond to identification with the BLS profession
Year 2-Growing confidence	Engagement in becoming a BLS	Increased exposure to laboratory practice and clinical training supported a clearer understanding of professional expectations. Participants emphasised competencies and professional values such as quality assurance, accuracy, analytical thinking, and responsible laboratory practice. For some participants, this increased exposure strengthened their understanding of and connection with the profession; for others, it provided a basis for questioning what the profession could offer as a future career
Year 2-Growing confidence	Engagement in becoming a BLS	Participants described developing confidence and a growing sense of suitability for the profession. Belonging appeared to develop through increased familiarity with professional practices and understanding of the responsibilities associated with the BLS role. However, engagement did not uniformly lead to belonging, as some participants remained uncertain about their future within BLS or considered alternative professional pathways
Year 2-Growing confidence	Engagement in becoming a BLS	Participants described BLS education as providing future opportunities. Some viewed the profession as one possible pathway that could lead to employment within the BLS field or provide skills transferable to other professional contexts. Others questioned the perceived range of career opportunities within BLS, suggesting that growing awareness could also generate uncertainty about long-term professional possibilities
Year 3 – Professional development	Alignment and belonging to the BLS	Participants’ understanding of the profession appeared to expand beyond laboratory tasks towards specialisation, expertise, and diverse career possibilities. Participants also connected the profession with contributing to healthcare and society. For some, this broader understanding supported a stronger connection with BLS; for others, it enabled them to recognise alternative ways of contributing to society outside healthcare
Year 3 – Professional development	Alignment and belonging to the BLS	Participants described a more developed understanding of BLS practice, including analytical reasoning, investigative approaches, and the interpretation of biological and pathological processes. The profession was increasingly understood through analytical and problem-solving perspectives. Greater understanding of actual working conditions could nevertheless challenge earlier expectations, particularly regarding autonomy, task variety, and the nature of everyday laboratory work
Year 3 – Professional development	Alignment and belonging to the BLS	Participants reflected on their professional preferences and possible future roles within different laboratory specialisations. Increased exposure to diverse areas of practice appeared to support a stronger connection with the BLS profession and emerging professional identity. At the same time, some participants reassessed their identification with BLS after encountering the realities of professional practice, demonstrating that increased exposure could lead not only to stronger belonging but also to professional distancing

### Imagination: imagining being a BLS

Participants described themselves as future BLS scientists in varied ways throughout their education. Their accounts reflected how they envisioned the profession, laboratory practice, career possibilities, and the contribution they hoped to make through their future work.

Early in the programme, some participants associated the profession with laboratory work and research. One Year 1 participant stated:

“*The BLS is quite good at lab work, and this is important if someone wants to do research.*”– Year 1, Peer Interview Group 1.

For other participants, the BLS programme represented a second choice or an alternative to healthcare programmes they did not wish to pursue:

” *It was the closest you could get to becoming a doctor. I wanted to become a doctor, but I didn’t get into the medical programme, and BLS was the closest option*” – Year 1, Peer Interview Group 1.

” *I wanted to get into a healthcare programme, but I didn't want to become a nurse… since I'm in clinical physiology, I thought I'd be able to do both laboratory work and work in a hospital, which would give me some freedom and flexibility*".

“*Jag vill komma in men jag vill inte bli sjuksköterska utan jag vill alltså, jag går klinisk fysiologi så tänkte både labba och jobba på sjukhus så har jag frihet eller flexibilitet*” – Year 1, Peer Interview Group 3.

These accounts show that the initial identification with the BLS profession was not necessarily grounded in a strong identification with the BLS community itself. Rather, the profession was initially understood in relation to another healthcare profession.

In Year 2 participants described an interest in laboratory practice as a motivation for choosing the programme:

“*I want to become a biomedical laboratory scientist because I love working in the lab.*”– Year 2, Peer Interview Group 3.

These accounts illustrate how the laboratory environment represented a central element of participants imagined professional development. For some participants, becoming a BLS was initially understood primarily through practical laboratory work and technical expertise. Participants also described visualizing themselves in professional laboratory settings. One Year 2 participant explained:

“*I'm such a visual person, and I visualize a lot of things. I can see myself in a lab environment. I picture myself in a lab wearing a white coat, with my glasses on, surrounded by a lot of machines, doing a lot of stuff*.”– Year 2, Peer Interview Group 3.

This description highlights how professional development was connected to the physical and material aspects of laboratory practice, including equipment, workplace environments, and professional appearance. Other participants imagined the profession as providing diverse career opportunities. One Year 2 participant described education as creating multiple future possibilities:

“*With this programme, it gives you such a solid foundation for so many different things—it opens so many doors. That’s a big part of why I wanted to become a BLS… I really enjoy working with that level of precision, where you become an expert in a specific area*.”– Year 2, Peer Interview Group 3.

Rather than identifying a single career pathway, this participant emphasized specialization, precision, and opportunities for continued professional development. Participants also connected their future professional development to contributing to healthcare. Although BLS work generally involves indirect rather than direct patient contact, students described helping others as an important motivation:

“*I have always thought that I want to work in healthcare. I want to help people and contribute to society, I think*.”– Year 2, Peer Interview Group 4.

These accounts suggest that participants incorporated the perceived societal value of laboratory diagnostics into their understanding of the profession. Other participants in the BLS educational programme did not necessarily envision a future within healthcare. Some participants expressed a desire to use their BLS competence in other socially oriented professions outsie healthcare, such as the police:

“*I want to apply to the police and work there”* Year 2, interview Group 1.

Rather than imagining a future within the healthcare community, this participant envisioned a role within policing, perhaps working in forensic biology or toxicology. This may indicate that the programme supports broader aspirations to contribute to society through professions beyond healthcare.

In Year 3, some descriptions of the profession included more specific aspects of BLS practice. One participant highlighted investigative work and contribution to diagnosis:

“*I've always enjoyed diagnostics and detective work with anatomy and diseases and stuff*.”– Year 3, Peer Interview Group 3.

Compared with earlier accounts that emphasized the laboratory environment, this description focused more on contribution to diagnostics by performing analyses and investigations. Together, these findings illustrate that imagination enabled participants to construct possible future professional development, although the ways in which they envisioned becoming BLSs varied between individuals.

### Engagement: engaging in becoming a BLS

Participants described engagement with the BLS profession through educational experiences, including theoretical learning, laboratory practice, and increasing familiarity with professional expectations. Within the CoP framework, engagement refers to participation in the practices of a community. Participants’ accounts indicated that this participation developed through different educational experiences and at different stages of the programme.

A Year 1 participant emphasized the importance of critical thinking in laboratory practice:

“*You should be critical to evaluate [laboratory] results in a meaningful way, to be able to perform an analysis according to a template, maybe*.”– Year 1, Peer Interview Group 1.

This account illustrates an early recognition that professional practice involves interpretation and evaluation, rather than solely performing technical procedures. At the same time, other participants expressed interest in the educational content while describing a stronger aspiration towards another healthcare profession:

“*I want to become a doctor. I want to get into the medical programme to continue studying, but I find the subject itself interesting. Studying it seems enjoyable, but perhaps not the profession itself to the same extent*” Year 1, Peer Interview Group 2.

This account adds an important dimension to engagement. The participant appeared to engage with the subject matter and educational activities while expressing less interest in the BLS profession as a future occupational professional development.

Several Year 2 participants described how their understanding of the profession developed through increased exposure to practical contexts. One participant reflected that early theoretical education made it difficult to understand the BLS role:

“*At the beginning of the education it’s almost all theory, it’s anatomy, its cell biology, it’s a lot of theory without finding out a lot about what the BLS actually does… we found out more now in the second year, and especially we will find out [more] during the work placement.*”– Year 2, Peer Interview Group 3.

This account highlights the importance of clinical placements and practical experiences in connecting theoretical knowledge with professional practice. Another Year 2 participant described developing a clearer understanding of professional expectations:

“*We have gained better clarity, like what is important as a BLS. I mean such as quality assurance, accuracy, analytical skill, and so on. It feels like we have been thoroughly trained.*”– Year 2, Peer Interview Group 2.

Rather than focusing only on technical competence, this participant emphasized broader professional values, including accuracy, quality assurance, and analytical ability. Participants highlight the perceived unknowingness of the BLS profession:

“*I think, or rather, something that you may have already encountered a little, but that you will encounter even more later, is that the profession is so unknown. When you talk to people, they ask, ‘What are you going to study?’ I say, ‘I’m going to study biomedical laboratory science,’ and they are like, ‘Excuse me?’ because no one knows what it is*” Year 2, Peer Interview Group 2.

These accounts illustrate another aspect of engagement: the unknowingness of BLS, both among people outside the profession and among participants themselves. The observation that “no one knows what it is” points to the profession’s limited visibility, while the reference to encountering it “a little” and then “much more later” suggests that education provided opportunities for engagement and familiarity with its professional role, practices, and significance.

In Year 3, participants described changes in both professional understanding and personal development. One participant reflected:

“*I’ve grown a lot… when it comes to responsibility and being analytical all the time. Not just accepting things at face value, but being critical and considering… And then, I’m starting to become stress-resistant*” – Year 3, Peer Interview Group 3.

This account illustrates how participation in the programme was associated with developing confidence, responsibility, and professional ways of thinking. Across the interviews, engagement was expressed through learning experiences, participation in laboratory education, and increasing familiarity with the practices and expectations associated with the BLS profession.

### Alignment: aligning with and belonging to the BLS

The theme of alignment describes how participants reflected on their relationship with the BLS profession and the extent to which they identified with its values, expectations, and future possibilities. Within the CoP framework, alignment refers to developing connections between personal goals and the practices of a professional community.

In Year 1, participants viewed BLS education as part of a broader professional pathway rather than a fixed destination. One participant explained:

“*Nothing is out of the question as a BLS. You can continue to study something else if you want… it’s kind of part of a pathway, and not the end goal, as a BLS*.”– Year 1, Peer Interview Group 2.

This account illustrates that professional development could include viewing BLS as one step within a broader career trajectory. In the peer groups from Year 2, participants described increasing confidence and belonging as they gained more practical experience. One participant stated:

“*I hope that I will be a good BLS and suited to the profession. I think it’s something that comes with time, and now that I have more clinical training, I’m learning increasingly and it’s becoming more natural*.”– Year 2, Peer Interview Group 4.

This reflects alignment as an ongoing process in which professional belonging developed through experience and growing familiarity with workplace practices. However, within the Year 2 interviews tensions in relation to alignment also appeared, as some participants negotiate, question, or even distance themselves from the BLS professional community:

“*It feels like there is a risk that there aren’t that many career opportunities. You become a BLS and get stuck, and then you might remain a BLS for 30 years. I’m just like, okay.*” Year 2, Peer Interview Group 2.

Rather than simply agreeing to adapt, the participant questions whether the BLS profession provides sufficient opportunities for movement, development, or alternative forms of participation. The idea of being “stuck” as a BLS suggests uncertainty about whether the professional community offers a desirable or sufficiently diverse future trajectory. While other participants negotiate the perceived position and value of the BLS profession in relation to other healthcare professions:

“*Or that we are replaceable, so to speak. But I don’t really think we are. It needs to be made clearer that we actually have expertise that other healthcare professions do not have*” Year 2, Peer Interview Group 2.

The contrast within this participant’s account between the perception that BLS professionals are “replaceable” and the assertion that they possess expertise other healthcare professions do not have illustrates how the distinctive contribution of the BLS professional community is articulated and defended. Another important dimension of alignment enhanced by other participants:

“*I feel that this is probably my biggest concern, really—that our profession will become so automated that it will become boring because you do so little yourself, and that certain things will be forgotten. But there are probably areas where this won’t happen. And then there is always the research side, where there are endless possibilities*. Year 2, Peer Interview Group 3.

The participant is evaluating whether future changes in BLS practice, particularly automation, are compatible with what they value in professional work. At the same time, the participant identifies research as an alternative space of professional possibility.

In Year 3, participants described a broader understanding of the diversity within the profession and how this influenced their own preferences. One participant reflected:

“*In the beginning, you hardly knew what a BLS did other than analyzing samples. Now you know all… the different clinical laboratories that exist, and which one is more to your taste than another. In my case, it’s microbiology, perhaps mainly bacteriology or parasitology*.”– Year 3, Peer Interview Group 1.

This account illustrates how increased exposure to different areas of laboratory practice supported participants in identifying professional interests and possible future roles. Other participants compared an imagined version of BLS practice, characterized by greater autonomy and variety, with the experienced reality of the workplace. The perceived discrepancy contributes to distancing from the professional role:

“*I don’t see myself as a BLS in the future. I thought we would have more autonomy in the workplace, that we would be able to do more, perhaps have more involvement in reporting results, and have more than just one method that you basically perform, or two that you alternate between. I thought we would be able to do everything, which in theory we should be able to, but in practice you end up performing one or two methods, and that didn’t really appeal to me*.” Year 3, Peer Interview Group 3.

This quotation demonstrates that greater exposure to professional practice does not necessarily result in stronger alignment. Instead, increased knowledge of the actual conditions of practice may lead a participant to reassess whether the profession corresponds with their expectations and aspirations.

Taken together, these findings illustrate how participants’ imagination, engagement, and alignment were reflected in their professional development across the three years of the programme.

## Discussion

This study explored BLS students’ professional development across three years of undergraduate education through Wenger’s [29, 30] CoP perspective. The findings suggest that professional development was not a straightforward progression from limited awareness in Year 1 to professional belonging in Year 3. Rather, students’ accounts demonstrated an ongoing and sometimes contradictory process involving imagination, engagement, and alignment. These modes of identification were evident across the three years, but they did not necessarily develop in parallel or lead to stronger identification with the BLS profession. Increased knowledge and participation could strengthen students’ understanding and sense of professional belonging, while at the same time revealing tensions between their expectations and the realities of professional practice. This finding is important because it challenges an overly linear understanding of professional development and supports viewing becoming a professional as an evolving process of participation, interpretation, and negotiation.

In the beginning of the programme, students’ accounts were strongly characterized by imagination. Participants imagined the BLS profession through laboratory environments, scientific knowledge, technical work, and possible future careers. This corresponds with Wenger’s [29, 30] conceptualization of imagination as a mode through which individuals construct possibilities for themselves by drawing on experiences, representations, and ideas about a community. For some students, imagining the BLS profession appeared to involve a relatively direct attraction to laboratory work, whereas others understood BLS in relation to other professions. One participant valued the subject matter but expressed a stronger aspiration to become a doctor. Another had desire to use tha BLS competence within the police. These accounts complicate the assumption that enrolment in professional education reflects an established professional commitment. The findings therefore suggest that imagination may involve both identification and distance. Students can imagine themselves within a profession while simultaneously imagining alternative professional futures. This is consistent with the broader literature indicating that professional development is influenced by students’ previous experiences, expectations, and interactions with professional environments rather than being produced solely through formal curriculum [11, 29, 30]. Importantly, the students’ uncertainty should not necessarily be interpreted as a lack of professional development. From a CoP perspective, imagining alternative futures may itself represent an active process of locating oneself in relation to professional communities. The findings consequently extend the understanding of imagination by showing that professional possibilities can include both movement towards and away from the BLS profession.

As students progressed through their studies, their accounts of their engagement indicated increasing exposure to laboratory education, practical activities, clinical training, and professional expectations. This corresponds with Wenger’s [11, 13–15, 23, 29, 30]. However, the findings also indicate that engagement was not simply equivalent to increased professional belonging.

One participant described the early programme as dominated by anatomy, cell biology, and other theoretical subjects, with limited understanding of what BLSs actually do. This suggests that participation in university education and participation in professional practice is not necessarily the same. Students may be highly engaged in academic activities while having limited access to the practices through which the professional role becomes meaningful. The transition towards clinical placement may therefore represent not merely an increase in practical experience but a change in participation. Through greater exposure to professional routines, responsibilities, quality assurance, accuracy, and analytical work, students can begin to connect theoretical concepts with their practical significance. This interpretation also supports the argument that clinical exposure alone does not automatically produce professional development.

The findings in our study suggest that engagement becomes professionally meaningful when students can understand the relationship between what they learn and how professional work is performed. Year 3 accounts concerning responsibility, critical thinking, and becoming more stress-resistant illustrates this broader development. These qualities extend beyond technical competence and correspond with research describing professional competence as encompassing communication, ethical practice, reflection, clinical reasoning, and professional behaviour [13, 14, 19]. Nevertheless, the findings demonstrate that engagement can also make previously implicit tensions more visible. Students’ increasing awareness of the BLS profession sometimes generated uncertainty about its visibility, career possibilities, autonomy, and future development. This is particularly evident in participants’ concerns that the profession was “unknown,” that they might become “stuck” as a BLS, or that automation could reduce the meaningfulness of their work. Such accounts are important because they demonstrate that professional learning involves not only acquiring the values and practices of a community but also evaluating whether those practices correspond with one’s own expectations and aspirations. Thus, engagement can create conditions for both professional attachment and critical questioning. This tension becomes particularly visible in the third mode, alignment.

Wenger [29, 30] describes alignment as connecting one’s activities and understanding with broader expectations and purposes within a community. In the present study, alignment was evident when students increasingly recognized the distinctive expertise of BLSs, identified areas of specialization, and considered how their work contributes to healthcare. The Year 3 participant who described microbiology, bacteriology, and parasitology as areas that were “more to their taste” illustrates how broader exposure to the profession could allow students to identify professional interests. In this sense, alignment was not simply alignment with BLS as a generic occupational category but increasingly involved understanding the diversity within the profession and locating one’s own preferences within it.

At the same time, alignment was not universal. Some participants explicitly questioned whether they could see themselves working as BLSs in the future. Similar uncertainty has been reported in nursing education. Marriott et al. [20] found that although clinical placement strengthened many students’ commitment to nursing, some remained ambivalent about their career choice. Canzan et al. [2], studying students who withdrew from nursing education, identified discrepancies between expectations of the profession and clinical reality, alongside doubts about suitability for nursing. These findings suggest that questioning one’s professional future is not unique to BLS education [2, 20]. The participant who expected greater autonomy and task variety but experienced work involving only one or two methods demonstrates how professional practice can challenge imagined versions of the profession. This finding is particularly significant for understanding professional development. Greater participation did not necessarily result in stronger alignment; instead, participation provided students with information that enabled them to reassess the relationship between their expectations and the actual conditions of practice. Professional development can therefore involve professional distancing as well as professional belonging. This finding is consistent with the broader CoP understanding that participation involves negotiation of meaning rather than passive adaptation to an established [29, 30]. Students are not simply socializing into becoming BLSs. They actively interpret professional practices and compare them with their own values, aspirations, and expectations. The findings indicate that students continuously negotiate their relationship with the profession. This is particularly relevant in BLS education because the profession may have lower public visibility than some other healthcare professions, as reflected in participants’ comments about others not knowing what BLS is.

Taken together, the findings suggest that imagination, engagement, and alignment overlap and interact throughout BLS education rather than constitute successive stages. Students imagined alternative professional futures while engaging in education, and engagement could strengthen professional connection or prompt questioning and distancing. Alignment involved negotiating professional practices in relation to students’ expectations, values, and future possibilities. Consistent with Wenger’s [29] understanding of learning as participation in social practice, these findings suggest that professional development involves acquiring competencies and making sense of them within professional contexts. Competence frameworks remain important for safe and effective practice but increasing certainty about entering the profession does not necessarily indicate development. Critical reflection, uncertainty, and distancing may also reflect a more informed understanding of professional practices and limitations.

The three-year trajectory can therefore be understood as an expansion of opportunities to encounter, interpret, and negotiate the BLS profession, with students developing different relationships to practice. For BLS education, this highlights the importance of environments that combine technical learning with exposure to professional settings, interaction with practitioners, feedback, and critical reflection. Professional development thus includes understanding what professional work means and considering whether, and how, students envisage participating in it. These processes warrant attention alongside competence assessment.

### Methodological considerations and contribution

The qualitative, interpretive design enabled an in-depth exploration of how BLS students constructed meanings of professional development within a specific educational context. Accordingly, the findings should be interpreted as contextually situated rather than as a generalizable developmental pathway [5]. The single-institution setting and voluntary recruitment strategy may have introduced self-selection bias, as students with greater engagement in their education or interest in professional development may have been more likely to participate. While these factors limit transferability, the findings offer theoretical insights that may be relevant to similar BLS educational contexts. The use of peer interviews represented both methodological strength and limitation. Conducting interviews between peers likely reduced hierarchical influences and encouraged open, reflective dialogue about educational experiences [8, 16, 21]. However, participants may also have moderated their responses to maintain positive peer relationships, and the absence of a researcher limited opportunities for probing, clarification, and exploration of divergent perspectives [24]. Furthermore, the first author's professional background as a BLS provided valuable disciplinary insight but also required continuous reflexive awareness of potential interpretive bias. To enhance credibility, data analysis was conducted through ongoing discussions within a multidisciplinary research team comprising both BLS and non-BLS researchers, allowing emerging interpretations to be critically examined from multiple perspectives [4, 22, 28]. This study contributes to the limited literature on BLS education by demonstrating how Wenger's [29, 30] modes of imagination, engagement, and alignment illuminate professional development as an iterative process of identity negotiation and belonging rather than a linear progression towards professional competence.

## Conclusion

This study explored BLS students’ professional development across three years of undergraduate education using Wenger’s CoP perspective. The findings demonstrate that professional development is an evolving and socially situated process rather than a linear progression towards professional competence or belonging. Imagination, engagement, and alignment provided complementary perspectives for understanding how students developed their knowledge, practices, values, and understanding of the BLS profession. While increased participation in educational and clinical contexts supported greater professional understanding and confidence for some students, it also enabled others to critically reassess their expectations and relationship with the profession. These findings highlight the importance of educational experiences that connect theoretical knowledge with authentic practice and provide opportunities for participation, reflection, and negotiation of professional meaning. Such development supports the development and application of competence in practice and, ultimately, contributes to the quality and safety of patient care. Future research should explore how different educational, organizational, and interprofessional contexts shape students’ trajectories of professional development and belonging throughout their education and into professional practice.

## Data Availability

The datasets generated and/or analysed during the current study are not publicly available due to the protection of participants’ confidentiality and ethical considerations. Further information regarding the data may be available from the corresponding author on reasonable request, subject to applicable ethical and confidentiality requirements.

## Appendix 1: English version of the interview guide

### Guidelines for conducting the interview

Before conducting the interview, please consider the following:

- Listen to each other.
- Ensure that both students have the opportunity to speak. Give each other space to contribute.
- The interview should take the form of a conversation and discussion between you and the other student.
- Allow sufficient time for reflection before responding.
- Do not judge or evaluate the answers.

### Interview questions

- Why do you want to become a biomedical laboratory scientist?
- How do you see yourself working as a biomedical laboratory scientist in the future?
- How have you developed so far in your journey toward becoming a biomedical laboratory scientist, and what do you need to develop further?
- What challenges have you encountered so far on your journey toward becoming a biomedical laboratory scientist, and what challenges do you think you will encounter in the future?
- How do you understand the concept of “accuracy” in relation to your future profession as a biomedical laboratory scientist?

### Submission of the interview recording

After completing the interview, participants were instructed to submit the audio recording to the designated researcher using the contact details provided to them separately.

## Abbreviations

BLS: Biomedical laboratory scientist
CoP: Communities of practice

## References

[1] Abunada T, Abdallah AM, Singh R, Abu-Madi M. Development and validation of a clinical practicum assessment tool for the NAACLS-accredited biomedical science program. Int J Environ Res Public Health. 2022;19(11):6651. 10.3390/ijerph19116651 PMC9180805 35682236

[2] Canzan F, Saiani L, Mezzalira E, Allegrini E, Caliaro A, Ambrosi E. Why do nursing students leave bachelor program? Findings from a qualitative descriptive study. BMC Nurs. 2022;21(1):71. 10.1186/s12912-022-00851-z PMC8966353 35351118

[3] Castillo-Page L, Bodilly S, Bunton SA. AM last page: Understanding qualitative and quantitative research paradigms in academic medicine. Acad Med. 2012;87(3):386. 10.1097/ACM.0b013e318247c660 22373638

[4] Cleland J, Durning SJ. Researching medical education. John Wiley & Sons; 2022.

[5] Cohen L, Manion L, Morrison K. Research methods in education. routledge; 2002.

[6] Contu A, Willmott H. Re-embedding situatedness: the importance of power relations in learning theory. Organ Sci. 2003;14(3):283–96.

[7] Edgren G. Developing a competence-based core curriculum in biomedical laboratory science: a Delphi study. Med Teach. 2006;28(5):409–17. 10.1080/01421590600711146 16973452

[8] Elliott E, Watson AJ, Harries U. Harnessing expertise: involving peer interviewers in qualitative research with hard-to-reach populations. Health Expect. 2002;5(2):172–8. 10.1046/j.1369-6513.2002.00158.x PMC5060135 12031057

[9] Farnsworth V, Kleanthous I, Wenger-Trayner E. Communities of practice as a social theory of learning: a conversation with Etienne Wenger. Br J Educ Stud. 2016;64(2):139–60.

[10] Fox S. Communities of practice, Foucault and actor-network therory. J Manag Stud. 2000;37(6):853–68.

[11] Frenk J, Chen L, Bhutta ZA, Cohen J, Crisp N, Evans T, et al. Health professionals for a new century: transforming education to strengthen health systems in an interdependent world. Lancet. 2010;376(9756):1923–58. 10.1016/S0140-6736(10)61854-5 21112623

[12] Graneheim UH, Lindgren B-M, Lundman B. Methodological challenges in qualitative content analysis: a discussion paper. Nurse Educ Today. 2017;56:29–34. 10.1016/j.nedt.2017.06.002 28651100

[13] Immonen K. Continuing professional development of medical laboratory professionals K. Immonen]. 2018.

[14] Immonen K, Oikarainen A, Tomietto M, Kääriäinen M, Tuomikoski A-M, Kaučič BM, et al. Assessment of nursing students’ competence in clinical practice: a systematic review of reviews. Int J Nurs Stud. 2019;100:103414. 10.1016/j.ijnurstu.2019.103414 31655385

[15] Judd BK, Scanlan JN, Alison JA, Waters D, Gordon CJ. The validity of a professional competence tool for physiotherapy students in simulation-based clinical education: a Rasch analysis. BMC Med Educ. 2016;16(1):196. 10.1186/s12909-016-0718-x PMC4974700 27492325

[16] Kallio H, Pietilä AM, Johnson M, Kangasniemi M. Systematic methodological review: developing a framework for a qualitative semi-structured interview guide. J Adv Nurs. 2016;72(12):2954–65. 10.1111/jan.13031 27221824

[17] Khan FMA, Moiz B, Rehman J, Majid H, Zeeshan M, Jafri L, et al. Advancing medical laboratory practice in Pakistan: insights from a focus group study on technologists’ training needs. BMC Med Educ. 2024;24(1):938. 10.1186/s12909-024-05836-x PMC11360743 39198837

[18] Khan M, Talbot A. CSMLS call to action: a medical laboratory science educator’s perspective. Cand J Med Lab Sci. 2019;81(2):27.

[19] Lejonqvist GB, Eriksson K, Meretoja R. Evaluating clinical competence during nursing education: a comprehensive integrative literature review. Int J Nurs Pract. 2016;22(2):142–51. 10.1111/ijn.12406 26369943

[20] Marriott PH, Weller-Newton JM, Reid KJ. Preparedness for a first clinical placement in nursing: a descriptive qualitative study. BMC Nurs. 2024;23(1):345. 10.1186/s12912-024-01916-x PMC11110182 38778354

[21] McIntosh MJ, Morse JM. Situating and constructing diversity in semi-structured interviews. Glob Qual Nurs Res. 2015;2:2333393615597674. 10.1177/2333393615597674 PMC5342650 28462313

[22] Nhalevilo EA, Ogunniyi M. Research as praxis: Perspectives on interpreting data from a science and indigenous knowledge systems project. African Journal of Research in Mathematics, Science and Technology Education. 2014;18(2):210–8.

[23] Noguera A, Robledano R, Garralda E. Palliative care teaching shapes medical undergraduate students’ professional development: a scoping review. Curr Opin Support Palliat Care. 2018;12(4):495–503. 10.1097/SPC.0000000000000402 30300153

[24] Payne-Gifford S, Brueton R, Hamilton G, Clark T, Chang YS, Parenting Science Gang Collective. Advantages and disadvantages of reciprocal peer-to-peer interviewing. Soc Res Pract. 2021;10:30–8.

[25] Persson B, Wilhelmsson M. Biomedicinsk analytiker-en profession att vara stolt över. Studentlitteratur AB; 2008.

[26] Roberts J. Limits to communities of practice. J Manag Stud. 2006;43(3):623–39.

[27] Stollenwerk MM, Gustafsson A, Edgren G, Gudmundsson P, Lindqvist M, Eriksson T. Core competencies for a biomedical laboratory scientist–a Delphi study. BMC Med Educ. 2022;22(1):476. 10.1186/s12909-022-03509-1 PMC9208704 35725406

[28] Swanwick T. Understanding medical education. Understanding Medical Education: Evidence, Theory, and Practice, 2018:1–6.

[29] Wenger E. Communities of practice: learning, meaning, and identity. Cambridge university press; 1999.

[30] Wenger E. Conceptual tools for CoPs as social learning systems: boundaries, identity, trajectories and participation. In: Social learning systems and communities of practice. Springer; 2010. p. 125–43.

